# Acknowledging unintended consequences of researching informal sperm donation networks: an opinion

**DOI:** 10.3389/fgwh.2024.1350640

**Published:** 2024-04-08

**Authors:** Lisa Jean Moore

**Affiliations:** SUNY Distinguished Professor of Sociology and Gender Studies, Purchase College, Purchase, NY, United States

**Keywords:** LGBT, donor insemination, reproduction, sperm, sperm banks, donors

## Introduction

I'll admit I was pretty excited when asked to write an opinion piece–I'm not often asked to give my opinion about things. Mostly I am working hard to *not* give my opinion, but rather, ground my scholarship in painstaking multi-year ethnographic research and feminist analysis followed up with extensive peer review. Beyond my sociological scholarship on human sperm (1, 2) and work as a Board President of a sperm bank, I've reproduced children using unregulated, non-clinical, informal sperm donation twice in the context of queer intimate relationships, once with a cis woman partner and once with a transgender man (as well as once using regulated, clinical donor sperm). I have first hand experience and very personal opinions about the practices of working outside of highly biomedicalized contexts and these are framed, of course, by my privilege and my exercising choice. The original request was made to encourage a piece that would cover any form of unregulated sperm donation that takes place outside of a clinic and be inclusive of marginalized people.

But before I began my manifesto, I wanted to clarify the assignment. I inquired to the editors if they were looking for any areas of opinion in particular. The reply included the editor's hopes to bring together some disparate literatures: in particular, non-clinical sperm donation in wealthy countries (predominantly, USA, UK, Australia), which, more recently, has typically taken place online, and sperm donation (or assisted conception practices more broadly) in low- and middle-income countries, and indigenous communities, which, may be less likely to rely on technology, and may draw more on informal donation ties or networks. There was a desire for a focus on women and other people who conceive, men and other people who donate, BIPOC, LGBT, working class, people. A bit daunted by this clarification (*perhaps the offer was too good to be true*), I began preliminary library research thinking that familiarizing myself with contemporary scholarship would help in offering my opinion.

## Analysis of literature

The germinal research of bioethicists Daniel Wikler and Norma Wikler from 1991 explores the demedicalization of insemination and bioethical implications of self insemination (3). The authors describe the informal networks of women emerging out of the women's liberation movement and feminist self-help health movement where single women and lesbians created networks for self insemination. The authors cite AIDS as a primary reason for the increased use of sperm banks that necessitated a move away from unregulated grapevines. And still they persisted. The occasional news story about unregulated sperm donation pops up here and there, such as the 2014 story from the US based NPR program *All Things Considered* (4). Listeners are introduced to VoyForum (https://www.voy.com/210024/) and The Known Donor (https://knowndonorregistry.com/) registry which both provide message board services for sperm donation, often free, between donors and recipients (both still currently active sites).

But what are the chief characteristics of unregulated sperm donation? There is scant but significant evidence-based scholarship on these informal, unregulated sperm donation practices but it is suggested that donor sperm is in short supply globally (specifically mentioned are UK, US, Denmark, and China) (5). Additionally we know the demand for black or other ethnic minority donor sperm is unmet (6). Research demonstrates that this lack of supply leads queer women of color to choose known sperm donors to avoid the white, heteronormative paradigms of the current cryobanking industry (7). Most significant for this essay, it is well established that the ability to gain access to regulated sperm, beyond this issue of homogeneity of supply, is not equally accessible among individuals. For example, the team led by Australian psychologist Damien Riggs reveals how non-heterosexual and/or gender non-normative individuals experience tremendous barriers for access to regulated, formal insemination pathways (8). In fact, there is a shortage of regulated local Australian sperm for all (heterosexual, non-heterosexual, and/or gender non-normative) individuals in Australia thus most sperm used now comes from the United States or Denmark. Presumably given extant institutional homophobia, transphobia and misogyny, some individuals may feel uncomfortable or discouraged by pursuing regulated sperm banks. If they believe these pathways are unavailable or restricted, they may achieve conception through other creative and inventive methods of networking.

These informal and unregulated networks require individuals to navigate the thorniness of deeply personal, intimate, and private engagements. Sociologist Petra Nordqvist has conducted extensive research on donor conception, families, and kinship. Her work, primarily conducted in England and Wales, demonstrates how informal sperm donation necessitates layers of complicated and intimate navigation of physical and emotional boundaries for lesbian couples and donors (9). Additionally, Nordqvist studying self-arranged conception argues that the non clinical, informal setting requires much negotiation about proposed parental involvement, health, and mechanics of conception (10). She further argues that in comparing the two types of seminal procurement the “legal distinction between the position of the donor in clinical and non-clinical conception added hurdles to non-clinical conception and increased the vulnerabilities and uncertainties for women who took this pathway (126).” If the people who take non-clinical pathways are already more vulnerable in stratified worlds, for example, they are lower income, gender nonconforming, single, or disabled, their access to clinical conception methods can be blocked. Therefore, people who are already more vulnerable are in the position to potentially experience increased vulnerability without clinical protection afforded regulated sperm recipients.

A team of Canadian social scientists interviewed 8 heterosexual men who donated sperm informally and they found that web-based assisted reproduction allowed more flexibility for these donors, yet it also required more careful negotiation of disclosure of this activity (11). Furthermore, a group of Canadian public health scholars characterized men who informally donate as ranking highly on scales of altruism; these donors cited personal agency, and desire for procreation as rationales for their participation (12). Behavioral economist Stephen Whyte describes informal donors from his Australian study as predominantly in committed relationships, identifying as other than heterosexual and embodying the personality trait of agreeableness (13).

Not unsurprisingly most of the research I have identified does not focus on low- and middle-income countries, nor indigenous communities. Rather it seems we know that even in high income countries, those who are more vulnerable by race, class, sex, gender, sexuality, and ability, seek out sperm donation in unregulated and non-clinical ways from donors who are likely partnered, nonheterosexual men who are altruistic and motivated to procreate. And still, it is very difficult to get an accurate estimate of how prevalent the practice of non-clinical, informal, and thus unregulated, sperm donation is. I turned to shaking the trees of my social networks. My friend, Megan, a doula and anthropologist replied, “It does sound like this editor is asking less for an opinion piece, and more for something that perhaps requires an ethnographer who wants to spend a year in a multisite comparison of sperm exchange practices globally.” Her reply, while validating my suspicions about the challenge of the assignment, also ignited in me the latent fear about qualitative research into subcultures, “hidden practices” or “data-deficient” social worlds.

## My opinion

This lack of scholarship led me to consider the role of social scientists and our fascination, almost preoccupation with revealing the social worlds of the understudied. In many ways, our profession has flourished on gaining entree, maintaining rapport, and collecting data from the most vulnerable members of our species (queer people, prisoners, sex workers, drug users, to name but a few). I believe many feel we are making social worlds knowable in order to reveal the matrices of oppression that humans endure to achieve well being or liveable lives. Perhaps we even believe we can create more liberation or solidarity through our scholarship. But what if our research pursuits are based in some naivete? Or even potentially harmful unintended consequences? What if there are no real tangible benefits to these individuals in revealing their practices of informal networks of sperm donation?

When imagining the design of a research project—a multi-sited ethnographic global study that oversamples for BIPOC, indigenous, or queer or gender nonconforming individuals who engage in non-regulated, informal semen donation—I'm reminded of the historical and ongoing debates in anthropology and the practice of academics “studying” indigenous cultures (14). As is often the case, scholars are not always mindful of relational accountability as explored in Shawn Wilson's indigenous research methods (15). We can extrapolate from indigenous methodological work questions such as: Do people actually want to be studied? If so, what do they want from an analysis of their communities? What could be gained from social scientific analysis of these worlds? Could our scholarly lens increase vulnerability for individuals or this entire meta-level practice?

As social scientists, we are moved to study the marginal; we make our careers on it. Are we making the marginal lives more unlivable by revealing practices to those regulatory bodies and state actors who wish to make them less possible? The regulatory gaze has rarely offered more expansive possibilities for people on the margins.

“In my opinion,” I texted my boyfriend, “I guess that people who get sperm who are queer or other ways marginalized are forced into informal ways of trying their best to have a life and make a family. They probably have no access to other ways to get sperm or can't get the type of sperm they want. And I get the sense that somewhere there is a hegemonic ideal that sperm should be regulated because it creates more “safety” (for whom, is presumably a very good question). And there is very little data from globally diverse places. Someone's dissertation will get published if they can reveal these worlds. But what if they might best be left to be hidden.”

Given the research we have, informal sperm donation being less well documented is perhaps useful, or even imperative, for those engaging in the process. Research might be used to reveal certain at-risk populations where regulation would diminish their ability to achieve conception. My concerns are beyond confidentiality to individual participants managed in IRB panels, rather I am deeply suspicious of ways we go about collecting data and the meta level analysis about the practices of informal, unregulated sperm donation. I perceive many recipients to be building a life carved out in entire practices of DIY, community-based craftiness.

Greater scrutiny brought by publishing social scientific studies could produce harm. While it does remain an empirical question if this research would create more barriers to who can conceive, I am concerned about the consequences. It is my opinion that progressive social change has rarely emerged from social science research that informs or enables state regulation. I do not believe that heteronormative, neoliberal, patriarchal, racist, ableist, biomedical capitalist states' policies produce greater liberation for the non-normative.
